# Using Bayh-Dole Act March-In Rights to Lower US Drug Prices

**DOI:** 10.1001/jamahealthforum.2024.3775

**Published:** 2024-11-01

**Authors:** Lisa Larrimore Ouellette, Bhaven N. Sampat

**Affiliations:** 1 Stanford Law School, Stanford, California; 2 Arizona State University Consortium for Science Policy and Outcomes, Washington, DC

## Abstract

**Importance:**

In December 2023, the Biden-Harris Administration released a proposed framework for exercising government march-in rights (effectively granting compulsory licenses for those patents to generic drug makers) under the Bayh-Dole Act on patents on taxpayer-funded drugs, which has renewed questions about whether march-in rights could promote cost savings through generic competition or harm pharmaceutical innovation.

**Objectives:**

To determine the feasibility of using march-in rights to remove patent barriers to generic competition.

**Design, Setting, and Participants:**

This cross-sectional study examined government funding information from multiple sources for patents listed in the Food and Drug Administration (FDA) Orange Book from 1985 to 2023. Data analysis was completed in August 2024.

**Exposures:**

New drug applications (NDAs) with Orange Book–listed patents, including (1) all new molecular entities (NMEs) approved from 1985 to 2022; (2) all NDAs with an Orange Book patent listed between 1985 and 2023; and (3) NDAs with unexpired patents listed in a recent (October 2023) edition of the Orange Book.

**Main Outcomes and Measures:**

The main outcome was whether the drugs had any or all patents that were public-sector patents subject to the Bayh-Dole Act, based on combining different data sources for identifying patents that resulted from federal funding. Public-sector patents resulting from intramural research, which are not subject to march-in rights under the Bayh-Dole Act, were identified separately.

**Results:**

Of 883 new molecular entities approved from 1985 to 2022, 68 (8%) had a Bayh-Dole patent, but only 18 (2%) had solely Bayh-Dole patents. Of 2832 drugs with patents listed for 1985 to 2023, 142 (5%) had a Bayh-Dole patent, but only 38 (1%) had solely Bayh-Dole patents. Of 1213 drugs with Orange Book patents listed in October 2023, 41 (3%) had a Bayh-Dole patent, but only 14 (1%) had solely Bayh-Dole patents.

**Conclusion and Relevance:**

This cross-sectional study found that, although Bayh-Dole march-in rights could remove patent barriers to generic entry for a few drugs, their overall effect would be limited.

## Key Points

**Question:**

What is the proportion of drugs for which the US government’s exercise of march-in rights could lower drug prices by removing patent barriers to generic competition?

**Findings:**

In this cross-sectional study of drugs approved by the Food and Drug Administration (FDA) with patents listed in the FDA’s Orange Book from 1985 to 2023, the share of drugs where all patents were subject to march-in was 2% for new molecular entities approved between 1985 and 2022, 1% for all new drug applications (NDAs) ever listing a patent in the Orange Book, and 1% for all NDAs on patents in 2023.

**Meaning:**

The findings suggest that few drugs have solely march-in–eligible patents, so the overall effect of march-in on removing patent barriers to competition would be limited.

## Teaser

This cross-setional study examines the feasibility of using march-in rights under the Bayh-Dole Act to systemically lower drug prices by removing patent barriers to competition using data from the Food and Drug Administration Orange Book

## Introduction

In 2023, as part of a suite of initiatives to lower pharmaceutical expenditures, the Biden-Harris Administration released a proposed framework for exercising government rights in taxpayer-funded drugs.^1,2^ In particular, if a drug’s price makes it inaccessible, the framework would allow agencies to exercise march-in rights (effectively granting compulsory licenses for those patents to generic drug makers) on public-sector patents. This proposal is the latest development in long-standing efforts to use march-in rights to improve access to medicines.^3^

The poster child for the potential effect of march-in rights is the prostate cancer drug Xtandi (enzalutamide), which can cost as much as $190 000 per year. All 3 of the patents for Xtandi were based on taxpayer-funded research at UCLA, under grants from the US Army and National Institutes of Health (NIH). In March 2023, the NIH refused a petition to march in on Xtandi patents, viewing the high list price as insufficient given that the drug was widely available to the public on the market.^4^ Concern over this decision spurred the Biden-Harris Administration’s proposal, under which price alone could justify marching in and facilitating generic entry.

March-in rights were established through the 1980 Bayh-Dole Act, which allows inventions created under federal grants to be patented. Rights retained by the federal government include the right under 35 U.S.C. § 203 to march-in to issue additional licenses to the patent “upon terms that are reasonable under the circumstances,” including “to alleviate health or safety needs.” There is considerable legal debate about whether march-in applies only in cases of failed commercialization^5,6^ or can be triggered by affordability considerations as well.^7^

March-in rights can be exercised only on patents covered by the Bayh-Dole Act, and can enable generic entry only if all unexpired patents on a drug are subject to the Bayh-Dole Act. March-in does not apply to privately funded inventions or to publicly funded inventions developed through intramural research such as at NIH labs (which are governed by the separate Stevenson-Wydler Act). They do not apply to all academic or university-owned patents,^8,9^ only those that were developed through government grants or contracts. March-in rights also do not apply to the large number of drugs for which the government contributed to drug development, when these contributions did not result in patents associated with the drug.^10,11,12^

We examine the feasibility of using march-in rights to systemically lower drug prices by removing patent barriers to competition using data from the Food and Drug Administration (FDA) Orange Book.^13^ We provide separate analyses for (1) NMEs approved by the FDA from 1985 to 2022, (2) the broader set of all new drug applications (NDAs) with patents in the Orange Book from 1985 to 2023, and (3) the subset of NDAs with patents listed in the October 2023 Orange Book. Compared with previous research on the government’s role in pharmaceutical innovation,^10,14,15^ we analyzed a broader array of drugs using more comprehensive data sources, we distinguished between drugs with any public-sector or all public-sector patents, and we provide separate analyses that excluded public-sector patents covered by the Stevenson-Wydler Act (and thus not subject to march-in rights) rather than the Bayh-Dole Act.

## Methods

### Orange Book Patent Data

We began by collecting patent data from the FDA Orange Book. Since the 1984 Hatch-Waxman Act, NDA applicants have been required to report patents covering these drugs to the FDA, which lists them in a document colloquially called the Orange Book. Since each version of the Orange Book includes only unexpired patents at the time of publication, we relied on a compilation of archival Orange Book listings from 1985 to 2016.^13^ We updated this dataset with Electronic Orange Book listings from 2017 to 2023. The study did not include human participants, and institutional review board approval was waived.

### FDA Compilation of NME Approvals

Previous analyses of the role of the public sector in drug innovation have often focused on the subset of NDAs corresponding to NMEs, or small-molecule drugs with active ingredients that have not previously been marketed as a drug in the US. We created a list of all NMEs approved from 1985 to 2022, drawn from a compilation by the FDA.^16^ Over this period, the FDA approved NDAs for 1002 NMEs. This data source also included information on the application number, approval year, applicant, and whether the approval was through the FDA’s priority review process, which we used as a proxy for clinical importance.^10^

### PatentsView Patent Data

We downloaded several files from the US Patent and Trademark Office (USPTO) PatentsView Database.^17^ The first file included the raw text of government-interest statements in all patents issued since 1976. The Bayh-Dole Act mandates that any party that enters a “contract, grant, or cooperative agreement” with a federal agency “for the performance of experimental, developmental, or research work” must include a statement in any resulting patents “specifying that the invention was made with Government support and that the Government has certain rights in the invention.” Second, the PatentsView assignee file allowed us to track patents where title is held by the NIH or other government agencies. For the small number of Orange Book patents issued before 1976 not covered by PatentsView, we manually reviewed the images at USPTO’s Public Patent Search website to collect this information.

### USPTO Certificates of Correction

Previous writing has found cases where a government-interest statement is not included when a patent issues but is added later in a postissuance certificate of correction.^14,18^ Unfortunately, certificates of correction are not available from PatentsView (or any other machine-readable database we know of) but are viewable in the image PDFs of patent documents. We obtained the USPTO certificate of correction Authority File, a listing of all patents with certificates of correction.^19^ We merged this file with all 9860 patents in the FDA Orange Books from 1985 to 2023. Of these, 3142 (32%) had a certificate of correction. We downloaded and digitized the full PDFs of each of these corrections. Then, we searched the certificate of corrections to find those that added government-interest statements.

### RePORTER/iEdison Data on NIH-Funded Patents

Since grantees sometimes fail to add government-interest statements to their patents,^20^ we also collected data from the NIH RePORTER database on all patents resulting from NIH-funded grants or contracts that were reported through the NIH iEdison system.^21^

### The Government Patent Register (Executive Order 9424 Conveyances)

In 1944, Executive Order 9424 created the Register of Government Interest in Patents to track government-funded patents. Modern register data are maintained in the USPTO Patent Assignment Database (UPAD). UPAD also includes data on patents reassigned to the government, which may pick up some government-assigned patents beyond those originally assigned to a government agency. Here we use the Gross and Sampat updated Government Patent Register,^22^ which consolidates the historical Government Register and UPAD information.

### Manual Review of University Patents

As a final safeguard, we reviewed at the USPTO Public Patent Search the images of all university-assigned patents that did not have government-interest statements (in PatentsView data or certificates of correction) and were not in RePORTER or the UPAD/Government Patent Register.

### Empirical Approach

We categorized as public-sector patents any Orange Book patent with a government-interest statement (in the original text, certificate of correction, or through manual review), with a government assignee, in NIH RePORTER, and/or with government rights or assignment per the UPAD/Register. We categorized as the Bayh-Dole patents the subset of public-sector patents excluding those for which the only evidence of public-sector interest was assignment to a government agency (either in original assignment or through assignments recorded in UPAD). Public-sector patents that are not Bayh-Dole patents are governed by the Stevenson-Wydler Act.

We categorized all 9860 Orange Book patents listed between 1985 and 2023. In patent-level analyses, we compared coverage of different sources in tracking public-sector patents. In drug-level analyses, we analyzed the scope for march-in for 3 sets of drugs: (1) the 883 patented NMEs approved from 1985 to 2022 (linked to 4497 unique patents), (2) the 2832 NDAs of any type (including not just NMEs) with patents in the Orange Book from 1985 to 2023 (linked to 9860 unique patents), and (3) for a snapshot on the scope for march-in rights for currently marketed drugs, the 1213 NDAs with patents in the October 2023 Orange Book (linked to 5633 unique patents). For each set of drugs, we calculated the number and percentage share of patents that were public-sector patents, and how these patents were captured by the various sources above. We also calculated the number and percentage share of each set of drugs with any public-sector patent and with all public-sector patents, and the same shares for Bayh-Dole patents, specifically, ie, public-sector patents excluding Stevenson-Wydler. Finally, for NMEs with mixed Bayh-Dole and other patents, we calculated the number of drugs where the Bayh-Dole patent is the last to expire, and the mean lag to expiration between non–Bayh-Dole and Bayh-Dole patents.

## Results

### Patent-Level Analyses

Table 1 reports the share of each set of Orange Book patents that are public-sector patents based on different data sources. These results illustrate the importance of combining multiple data sources to identify public-sector involvement in drug patenting. For example, of the 4497 unique patents linked to NMEs approved between 1985 and 2022, 139 (3.1%) have a government-interest statement in the original patent text, whereas an additional 24 (for 163 total [3.6%]) have a government-interest statement added via a certificate of correction.

**Table 1.** Orange Book Patents Resulting From Public-Sector Funding by Source of Information on Public-Sector Funding^a^

Source of patent information	Patents, No. (%)		
	All NMEs approved 1985-2022	All NDAs in 1985-2023 Orange Books	October 2023 Orange Book
Total	4497	9860	5633
Government-interest statement	139 (3.1)	198 (2.0)	109 (1.9)
Government-interest statement (with CoC)	163 (3.6)	230 (2.3)	123 (2.2)
NIH RePORTER	100 (2.2)	129 (1.3)	74 (1.3)
RePORTER (no GI)	24 (0.5)	28 (0.3)	13 (0.2)
RePORTER (no GI including CoC)	1 (0)	1 (0)	0
GI statement (with CoC) but not in RePORTER	64 (1.4)	102 (1)	49 (0.9)
Government assignee (Stevenson-Wydler patents)	22 (0.5)	31 (0.3)	11 (0.2)
Register/UPAD	95 (2.1)	115 (1.2)	51 (0.9)
Register/UPAD only	1 (0)	4 (0)	1 (0)
Overall: public-sector patents	184 (4.1)	260 (2.6)	131 (2.3)

Abbreviations: GI, Government Interest Statement in Patent; CoC, certificate of correction; NIH, National Institutes of Health; NME, new molecular entity; Register/UPAD, Government Patent Register/US Patent and Trademark Office Assignment Database.

^a^ Stevenson-Wydler patents are those assigned to a US government agency. The percentages do not add to 100 since a patent can be listed in multiple sources. Overall public-sector patents are those listed in one or more of the sources. The shares are taken across 3 different sets of patents: those listed in the Orange Book (from 1985-2023) for NMEs approved from 1985 to 2022, those listed for any NDAs (new drug applications) in Orange Books from 1985 to 2023, and those listed for any NDAs in the October 2023 Orange Book. NMEs are new molecular entities, a particular type of new drug application with active ingredients where no active moiety was previously approved by the Food and Drug Administration.

Of the 100 patents linked to NMEs that are listed in the NIH RePORTER database, 24 had no government-interest statement in the original text, but only 1 was missing a government-interest statement when Certificates of Correction are considered. Given the difficulty accessing information in Certificates of Correction, RePORTER data thus remains important for capturing the full set of public-sector patents. However, RePORTER data were also incomplete for NMEs; there were 64 patents with government-interest statements not included in RePORTER. Reviewing the missing patents by hand, we found that of these some were from other agencies, but most reflected NIH grants and should have been included in RePORTER.

Searching for patents with government assignees yielded 22 public-sector patents covered by the Stevenson-Wydler Act for the NMEs, which are relevant for measuring public-sector involvement but not for Bayh-Dole march-in rights. Finally, the Register/UPAD dataset yielded 1 NME-linked patent that was not listed in any of the other data sources. Reviewing the UPAD records revealed that this was a “corrective assignment” (to the Department of Veterans Affairs), not a 9424 conveyance, so we treated it as a public-sector patent but not a Bayh-Dole patent. Overall, 184 of the 4497 unique NME-linked patents (4.1%) were public-sector patents, based on information from all sources. Table 1 shows similar trends for the 9860 unique patents linked to all NDAs with Orange Book patents from 1985 to 2023 (with a total of 2.6% being public-sector patents), and for the 5633 unique patents linked to NDAs in the October 2023 Orange Book (with 2.3% being public-sector patents). Together, these results suggest that the different data sources have complementary information about public-sector patents.

To illustrate the problem of underreporting of government interest in original patent filings, eTable 1 in Supplement 1 lists all 32 Orange Book patents (from the full set of 9860) that provide government interest statements only via certificates of correction. The lag between filing the patent and issuing the correction can be considerable, including for high-profile, blockbuster drugs. For example, Lyrica (pregabalin) had a patent filed in 1995 with the government interest added via a certificate of correction 8 years later in 2003; Prozac (fluoxetine hydrochloride) had a patent filed in 1988 with the government interest added via a certificate of correction 13 years later in 2001; and Gleevec (imatinib mesylate) had a patent filed in 2003 with the government interest added via a certificate of correction 16 years later in 2019. The extent of these lags and the fact that Certificates of Correction are only accessible through image files of patent documents makes it challenging to fully evaluate the public sector’s role in drug development through government-interest statements alone.

### Drug-Level Analyses

Table 2 reports our main analyses, at the drug level for drugs with at least 1 Orange Book patent. Overall, 9.1% of NMEs approved from 1985 to 2022 had at least 1 public-sector patent. The share was more than twice as high for priority-review drugs (13%) compared with standard-review drugs (5.7%), consistent with previous research.^10,14^ Focusing on NMEs (drugs with new active ingredients) or just priority-review NMEs (drugs that offer significant improvements in safety or effectiveness) provides 2 measures of the most important drugs. When we instead examine all NDAs with Orange Book listings, not just NMEs, the percentage with any public-sector patent is 5.9% for the full 1985 to 2023 dataset, and 3.7% for NDAs in the October 2023 Orange Book.

**Table 2.** Patented Orange-Book Drugs With Any Public-Sector Patents and All Public-Sector Patents^a^

Variable	Total No.	No. (%)			
		Public		Bayh-Dole Act	
		Any	All	Any	All
All NMEs approved 1985-2022	883	80 (9.1)	22 (2.5)	68 (7.7)	18 (2)
Standard review	474	27 (5.7)	6 (1.3)	26 (5.5)	6 (1.3)
Priority review	409	53 (13)	16 (3.9)	42 (10.3)	12 (2.9)
All NDAs listed in 1985-2023 Orange Book	2832	167 (5.9)	46 (1.6)	142 (5)	38 (1.3)
All NDAs listed in October 2023 Orange Book	1213	45 (3.7)	16 (1.3)	41 (3.4)	14 (1.2)

Abbreviations: NDA, new drug applications; NME, new molecular entity.

^a^ The first 2 results columns include all public-sector patents, the last 2 focus on Bayh-Dole patents, excluding public-sector patents that are assigned to government agencies (eg, National Institutes of Health) that would be governed by the Stevenson-Wydler Act rather than the Bayh-Dole Act, and thus not subject to Bayh-Dole Act march-in rights. NMEs are new molecular entities, a particular type of NDA with active ingredients where no active moiety was previously approved by the Food and Drug Administration (FDA). We focused only on NMEs with an Orange Book patent listing between 1985 and 2023. Priority NMEs are those that received priority FDA review; standard NMEs are those that received standard FDA review. For the NME sample, χ^2^ tests of independence revealed the difference between priority and standard review drugs was statistically significant at the 5% level for drugs with any public sector patents (*P* < .001), all public-sector patents (*P* = .01), any Bayh-Dole (*P* = .01), but not for all Bayh-Dole (*P* = .08).

The share of drugs with only public-sector patents is lower. For the NMEs, only 2.5% have all public-sector patents. Again, the share is higher for priority-review drugs than for standard-review drugs (3.9% vs 1.3%). For the NDAs, 1.6% of the full dataset of Orange Book patents and 1.3% of the October 2023 snapshot have all public-sector patents.

Examining all public-sector patents (both Bayh-Dole and Stevenson-Wydler Act) is useful both for comparing with previous results that have not distinguished the two, and because the public sector may have some rights and points of leverage for Stevenson-Wydler patents beyond march-in rights. However, to enable generic competition through march-in rights alone, all unexpired patents must be public-sector patents subject to the Bayh-Dole Act, ie, excluding Stevenson-Wydler patents. The third column of Table 2 reports the share of drugs with any Bayh-Dole patents: 7.7% of NMEs (10.3% for priority-review vs 5.5% for standard-review), 5% of the full dataset of NDAs, and 3.4% of NDAs with patents in the October 2023 Orange Book. The final column of Table 2 reports the share of drugs with only Bayh-Dole patents: 2% of NMEs (2.9% for priority-review vs 1.3% for standard review), 1.3% of all NDAs, and 1.2% of NDAs on-patent in October 2023. The October 2023 Orange Book provides a snapshot of current drugs, illustrating that march-in rights could enable generic competition for only 14 of 1213 NDAs that were on-patent at that time. eTable 2 in Supplement 1 lists NMEs with all Bayh-Dole patents, and eTable 3 in Supplement 1 lists NMEs with both Bayh-Dole and other patents. eTable 4 in Supplement 1 lists all NDAs with at least a Bayh-Dole patent, along with the share of listed patents for that NDA that are Bayh-Dole patents.

### Expiration Dates of Bayh-Dole Patents vs Other Patents

Although the overall share of drugs with only Bayh-Dole patents is low, march-in rights could still be useful for drugs with a mix of Bayh-Dole and other patents if the Bayh-Dole patents were last to expire. To evaluate how often this is true, we calculated the lag between the last-to-expire Bayh-Dole patent and the last-to-expire non–Bayh-Dole patent for all 50 NMEs with mixed patent portfolios listed in eTable 3 in Supplement 1 (using Orange Book data on expiration dates). A Bayh-Dole patent expires last for only 6 of these 50 drugs. For these 6 drugs, the mean (SD) time between the last non–Bayh-Dole patent expiring and the last Bayh-Dole Act patent expiring is 5.8 (2.9) years, which is the amount by which exercising march-in rights could speed the removal of patent barriers for this handful of drugs. In general, however, Bayh-Dole patents expire earlier than other patents, so marching in on all Bayh-Dole patents would leave later-expiring non–Bayh-Dole patents. For the full set of 50 drugs, the mean time between the last Bayh-Dole patent expiring and the last non–Bayh-Dole patent expiring is 5.9 (7.2) years.

### March-In Rights, Patent Challenges, and Secondary Patents

Although marching in on Bayh-Dole patents would still leave later-expiring non–Bayh-Dole patents for most drugs with both types of patents, march-in rights could still help facilitate generic competition if the remaining nonpublic patents were vulnerable to removal through other policy tools. The 1984 Hatch-Waxman Act created a mechanism—paragraph IV patent challenges—allowing potential generic entrants to challenge Orange Book–listed patents as invalid or noninfringed. Compared with primary patents on a drug’s active ingredient, secondary patents—such as patents on the formulation, method of use, or metabolites—have been shown to be more vulnerable to being deemed invalid or noninfringed under these challenge procedures.^23,24,25^ March-in rights could thus have broader scope for drugs where the public sector has rights to the primary patents, and other entities hold only secondary patents. Beyond patent challenges, the Biden-Harris administration has promoted various other approaches to curtailing secondary patents, including improved FDA-USPTO cooperation and FTC scrutiny of potentially improper Orange Book patent listings. Coding the patents in our dataset as primary or secondary would be a valuable avenue for future work.

## Discussion

The findings of this study suggest that march-in rights could apply to only a few drugs. We estimate that 8% of the 883 NMEs approved from 1985 to 2022 have at least 1 Bayh-Dole patent. The share was more than twice as high for priority-review drugs (10%) compared with standard-review drugs (5%). However, march-in rights can enable generic entry only if all unexpired patents on a drug are subject to the Bayh-Dole Act. This is true for only 18 drugs in the 1985 to 2022 NME sample, about 2% of the 883 drugs. Xtandi, the poster child for march-in rights, is one of these. However, our analyses show that Xtandi is the exception, not the rule, in the government’s ability to use march-in rights.

### Limitations

This study has limitations. One limitation is that this study focused on small-molecule pharmaceuticals. We did not directly look at biologic drugs—disproportionately represented among high-cost drugs—due to the lack of public data about which patents cover these drugs. Because there tends to be even more patents per drug for biologics,^26^ our hypothesis is the share of biologics with all public-sector patents would likely be even smaller than for small-molecule drugs. We also did not directly look at sales for the drugs in our sample, limiting what we can directly say about the overall impact on expenditures or the differential impact on more lucrative drugs. Previous research suggests the propensity to accumulate secondary patents on drugs, a practice sometimes called “evergreening,” is more common for higher-sales drugs.^23,24^ Thus we would expect the share of drugs with all public-sector patents to be lower for more lucrative drugs, which would limit the impact of march-in rights on expenditures. Finally, as noted above, coding whether Bayh-Dole patents are stronger primary patents and non–Bayh-Dole patents are weaker secondary patents is an important topic for future research.

Our analyses also suggest considerable underreporting in any individual source of public-sector patents. Although combining multiple sources ameliorates this, we do not know the share of patents not reported anywhere. Policies promoting better compliance in reporting, or empirical approaches to better assess these unknown unknowns seem important.

## Conclusions

The results of this cross-sectional study suggest that the overall impact of exercising Bayh-Dole march-in rights on removing patent barriers to competition would be small. In our snapshot of currently marketed drugs with patents in the October 2023 Orange Book, all patents are subject to march-in rights for only 14 of 1213 drugs (1.2%). Even if all non–Bayh-Dole patents are weaker secondary patents that could be successfully challenged under paragraph IV procedures, this would only increase the number of drugs for which march-in rights are relevant to 41 (3.4%). March-in rights are not relevant for most drugs.

If policymakers want to lower prices for a broader array of drugs, they should thus focus on other policies. For example, drug pricing policies that do not depend on whether a drug benefitted from federal funding include allowing drug production by or for the US under 28 U.S.C. § 1498,^27^ price setting through government insurance like Medicare and Medicaid,^28^ or directly purchasing patented products as for COVID-19–related technologies.^29^ Experimenting with more proactive drug access policies in the initial licensing of both Bayh-Dole patents and Stevenson-Wydler patents may also be worthwhile.^30^

## Notes

Supplement 1. eTable 1. Orange Book Patents Where Government-Interest Statements Were Added Via Certificates of Correction eTable 2. FDA-Approved New Molecular Entities (1985-2022) with all Bayh-Dole Patents eTable 3. FDA-Approved New Molecular Entities (1985-2022) with Bayh-Dole and Other Patents eTable 4. FDA-Approved New Drug Applications with at Least One Bayh-Dole Patent in the Orange Book (editions 1985-2023), with the Share of Bayh-Dole Patents

Supplement 2. Data Sharing Statement
