# Supplementary material for: Using Bayh-Dole Act March-In Rights to Lower US Drug Prices
Source: JAMA Health Forum. 2024 Nov 1;5(11):e243775. doi: 10.1001/jamahealthforum.2024.3775 (PMC11530938; doi:10.1001/jamahealthforum.2024.3775)
Supplement: Supplement 1. — eTable 1. Orange Book Patents Where Government-Interest Statements Were Added Via Certificates of Correction eTable 2. FDA-Approved New Molecular Entities (1985-2022) with all Bayh-Dole Patents eTable 3. FDA-Approved New Molecular Entities (1985-2022) with Bayh-Dole and Other Patents eTable 4. FDA-Approved New Drug Applications with at Least One Bayh-Dole Patent in the Orange Book (editions 1985-2023), with the Share of Bayh-Dole Patents [file jamahealthforum-e243775-s001.pdf]

Supplemental Online Content

Ouellette LL, Sampat BN. Using Bayh-Dole Act march-in rights to lower US drug prices. *JAMA Health Forum*. 2024;5(11):e243775. doi:10.1001/jamahealthforum.2024.3775

eTable 1. Orange Book Patents Where Government-Interest Statements Were Added Via Certificates of Correction

eTable 2. FDA-Approved New Molecular Entities (1985-2022) with all Bayh-Dole Patents

eTable 3. FDA-Approved New Molecular Entities (1985-2022) with Bayh-Dole and Other Patents

eTable 4. FDA-Approved New Drug Applications with at Least One Bayh-Dole Patent in the Orange Book (editions 1985-2023), with the Share of Bayh-Dole Patents

This supplemental material has been provided by the authors to give readers additional information about their work.

© 2024 Ouellette LL et al. *JAMA Health Forum.*

© 2024 Ouellette LL et al. *JAMA Health Forum.*

eTable 1. Orange Book Patents Where Government-Interest Statements Were Added Via Certificates of Correction

NDA	Drug	Patent	In RePORTER?
18312	CALDEROL (calcifediol)	3565924	No
18936	PROZAC (fluoxetine hydrochloride)	4971998	Yes
19937	ADENOCARD (adenosine)	4673563	Yes
20408	TRUSOPT (dorzolamide hydrochloride)	4619939	Yes
20898	THYROGEN (thyrotropin alfa)	6114144	No
20898	THYROGEN (thyrotropin alfa)	6365127	Yes
20919	GEODON (ziprasidone mesylate)	5376645	No
20919	GEODON (ziprasidone mesylate)	5134127	No
21320	PLENAXIS (abarelix)	6455499	Yes
21320	PLENAXIS (abarelix)	6423686	Yes
21335	GLEEVEC (imatinib mesylate)	6958335	Yes
21446	LYRICA (pregabalin)	5563175	Yes
21487	NAMENDA (memantine hydrochloride)	5614560	Yes
21674	MENOSTAR (estradiol)	6692763	Yes
21746	SURFAXIN (lucinactant)	5407914	Yes
22468	FOLOTYN (pralatrexate)	6028071	Yes
22468	FOLOTYN (pralatrexate)	8299078	No
203137	VIZAMYL (flutemetamol f-18)	7270800	Yes
203137	VIZAMYL (flutemetamol f-18)	8691185	Yes
203137	VIZAMYL (flutemetamol f-18)	8236282	Yes
206488	EXONDYS 51 (eteplirsen)	8486907	Yes
206488	EXONDYS 51 (eteplirsen)	9018368	Yes

© 2024 Ouellette LL et al. *JAMA Health Forum.*

208627	TPOXX (tecovirimat)	7737168	Yes
209531	SPINRAZA (nusinersen sodium)	8361977	Yes
210565	INVELTYS (loteprednol etabonate)	9056057	Yes
210933	EYSUVIS (loteprednol etabonate)	10857096	Yes
210951	ERLEADA (apalutamide)	8445507	Yes
211970	VYONDYS 53 (golodirsen)	9994851	Yes
211970	VYONDYS 53 (golodirsen)	9024007	Yes
213026	AMONDYS 45 (casimersen)	8524880	Yes
213026	AMONDYS 45 (casimersen)	9447415	Yes
214793	PYLARIFY (piflufolastat f-18)	9861713	Yes

Notes: Some Orange Book patents can link to multiple drugs. The drug name listed here corresponds to the first new drug application for each patent.

© 2024 Ouellette LL et al. *JAMA Health Forum.*

eTable 2. FDA-Approved New Molecular Entities (1985-2022) with all Bayh-Dole Patents

NDA	Drug	Bayh-Dole Patents (U.S. Patent No.)
19530	Ucephan (sodium phenylacetate, sodium benzoate)	4284647
19836	Supprelin (histrelin acetate)	4244946
19863	Geref (sermorelin acetate)	4517181, 4703035
19937	Adenocard (adenosine)	4673563
20044	Exosurf (colfosceril palmitate, cetyl alcohol, tyloxapol)	4312860, 4826821, 5110806
20084	Iobenguane Sulfate I 131 (iobenguane sulfate i 131)	4584187
20412	Zerit (stavudine)	4978655
21500	Emtriva (emtricitabine)	5210085, 5814639, 5914331, 6642245, 6703396, 7402588
21673	Clolar (clofarbine)	4918179, 5384310, 5661136
21746	Surfaxin (lucinactant)	5407914
22253	Vimpat (lacosamide)	5654301, RE38551
22468	Folotyn (pralatrexate)	6028071, 7622470, 8299078
202008	Amyvid (florbetapir f 18)	7687052, 8506929
203415	Xtandi (enzalutamide)	7709517, 8183274, 9126941
204677	Neuraceq (florbetaben f 18)	7807135
208627	Tpoxx (tecovirimat)	7737168, 8039504, 8124643, 8530509, 8802714, 9339466
211996	Vyndaqel (tafamidis meglumine)	7214695, 7214696, 8168663, 8653119
214375	Xenoview (xenon xe 129 hyperpolarized)	10583205, 11052161

© 2024 Ouellette LL et al. *JAMA Health Forum.*

eTable 3. FDA-Approved New Molecular Entities (1985-2022) with Bayh-Dole and Other Patents

NDA	Drug	Bayh-Dole Patents	Other Patents
18936	Prozac (fluoxetine hydrochloride)	4035511, 4083982, 4971998	4018895, 4194009, 4314081, 4329356, 4590213, 4594358, 4626549, 4647591, 4683235, 5114976, 5744501, 6960577
19785	Cardiolite (technetium tc-99m sestamibi)	4452774	4885100, 4894445, 4988827, 5324824
19829	Ceretec (technetium tc-99m exametazime)	4615876	4789736
19880	Paraplatin (carboplatin)	4140707	4657927
20212	Zinecard (dexrazoxane)	5242901	4275063, 4963551
20408	Trusopt (dorzolamide hydrochloride)	4619939	4797413
20451	Photofrin (porfimer sodium)	4649151, 4866168, 4932934, 5028621, 5145863	5438071
20597	Xalatan (latanoprost)	4599353	5296504, 5422368, 6429226, 7163959
20659	Norvir (ritonavir)	5541206, 5635523, 5648497, 5674882, 5846987, 5886036	5484801, 5948436, 6037157, 6703403
20819	Zemplar (paricalcitol)	5246925, 5587497, 5597815	6136799, 6361758
20845	Inomax (nitric oxide)	5485827, 5873359	5558083, 5732693, 5752504, 6125846, 8282966, 8291904, 8293284, 8431163, 8573209, 8573210, 8776794, 8776795, 8795741, 8846112, 9265911, 9279794, 9295802, 9408993, 9770570
21119	Visudyne (verteporfin)	5798349	4833790, 4883790, 4920143, 5095030, 5214036, 5283255, 5707608, 5756541, 5770619, 6074666
21197	Cetrotide (cetrorelix acetate)	4800191, 5198533	6319192, 6863891, 7605121
21226	Kaletra (lopinavir, ritonavir)	5541206, 5635523, 5648497, 5674882, 5846987, 5886036	5914332, 5948436, 6037157, 6232333, 6284767, 6458818, 6521651, 6703403, 7141593, 7432294
21320	Plenaxis (abarelix)	5843901, 6423686, 6455499	5968895, 6180608, 6699833
21335	Gleevec (imatinib mesylate)	6958335	5521184, 6894051, RE43932

© 2024 Ouellette LL et al. *JAMA Health Forum.*

NDA	Drug	Bayh-Dole Patents	Other Patents
21366	Crestor (rosuvastatin calcium)	7030152, 7964614	6316460, 6589959, 6858618, RE37314
21446	Lyrica (pregabalin)	5563175, 6197819	6001876, RE41920
21481	Fuzeon (enfuvirtide)	5464933	6133418, 6475491
21487	Namenda (memantine hydrochloride)	5614560	5061703
21773	Byetta (exenatide)	5424286	6858576, 6872700, 6902744, 6956026, 7297761, 7521423, 7741269
21964	Relistor (methylnaltrexone bromide)	6559158	8247425, 8420663, 8552025, 8822490, 9180125, 9492445, 9669096, 10376584
21991	Zolinza (vorinostat)	7399787, 7456219, 7652069, 7732490, 7851509, 8067472, 8101663	6087367, 8093295, 8450372, RE38506
21995	Januvia (sitagliptin phosphate)	6890898, 7078381, 7459428	6303661, 6699871, 7125873, 7326708
22271	Nesina (alogliptin)	6890898, 7078381, 7459428	6150383, 6211205, 6303640, 6303661, 6329404, 7807689, 8173663, 8288539, 8697125
201280	Tradjenta (linagliptin)	6890898, 7078381, 7459428	6303661, 7407955, 8119648, 8178541, 8673927, 8846695, 8853156, 8883805, 9173859, 9486526, 10034877, 11033552
202207	Lymphoseek (technetium tc 99m tilmanocept)	6409990	9439985
203100	Stribild (elvitegravir, cobicistat, emtricitabine, tenofovir disoproxil fumarate)	5814639, 5914331, 6642245, 6703396	5922695, 5935946, 5977089, 6043230, 7176220, 7635704, 8148374, 8592397, 8633219, 8716264, 8981103, 9457036, 9744181, 9891239, 10039718
203137	Vizamyl (flutemetamol f 18)	7270800, 8236282, 8691185	7351401, 8916131
205494	Cerdelga (eliglustat)	6916802, 7253185	7196205, 7615573, 10888544, 10888547, 11458119
206488	Exondys 51 (eteplirsen)	8486907, 9018368, 10781451, RE47769, RE47751	9243245, 9416361, 9506058, 10337003, 10364431, 10533174, RE48468
207561	Genvoya (elvitegravir, cobicistat, emtricitabine, tenofovir alafenamide)	5814639, 5914331, 6642245, 6703396	7176220, 7390791, 7635704, 7800788, 7803788, 8148374, 8633219, 8754065, 8981103, 9296769, 9891239, 10039718

© 2024 Ouellette LL et al. *JAMA Health Forum.*

NDA	Drug	Bayh-Dole Patents	Other Patents
207924	Olumiant (baricitinib)	9737469	8158616, 8420629, 9089574, 11045474
208054	Axumin (fluciclovine f 18)	5808146	9387266, 10010632, 10124079, 10716868, 10933147, 10953112, 10967077
209531	Spinraza (nusinersen)	7838657, 8110560, 8361977, 10266822	6166197, 6210892, 7101993, 8980853, 9717750, 9926559, 10436802
209776	Vabomere (meropenem and vaborbactam)	11376237	8680136, 9694025, 10172874, 10183034, 10561675, 11007206
209899	Zeposia (ozanimod)	8481573, 8796318, 9382217	10239846
210251	Biktarvy (bictegravir, emtricitabine, tenofovir alafenamide)	6642245, 6703396	7390791, 7803788, 8754065, 9216996, 9296769, 9708342, 9732092, 10385067, 10548846, 11744802
210450	Orilissa (elagolix)	6872728, 7056927, 7176211, 7179815, 7419983, 7462625	10537572, 10682351, 11344551, 11542239, 11690845, 11690854, 11707464
210557	Vyleesi (bremelanotide)	6579968	6794489, 9352013, 9700592, 10286034, 11590209
210922	Onpattro (patisiran)	8552171, 9193753	8058069, 8158601, 8168775, 8334373, 8362231, 8372968, 8492359, 8642076, 8741866, 8778902, 8802644, 8822668, 8895718, 8895721, 9234196, 9364435, 9567582, 9943538, 9943539, 10240152, 11079379, 11141378
210951	Erleada (apalutamide)	8445507, 8802689, 9388159, 9987261	9481663, 9884054, 10052314, 10702508, 10849888, RE49353
211109	Xerava (eravacycline)	10961190, 11578044	8796245, 8906887
211970	Vyondys 53 (golodirsen)	9024007, 9994851, 10227590, 10266827, 10421966, 10968450, 10995337, RE47691	9416361, 10533174
213026	Amondys 45 (casimersen)	8524880, 9447415, RE48960	9228187, 9416361, 9758783, 10287586, 10533174, 10781450
213378	Lybalvi (olanzapine and samidorphan)	7262298, 7956187, 8252929	8778960, 9119848, 9126977, 9517235, 10300054,

© 2024 Ouellette LL et al. *JAMA Health Forum.*

NDA	Drug	Bayh-Dole Patents	Other Patents
			10716785, 11185541, 11241425, 11351166, 11707466
214012	Leqvio (inclisiran)	10590418	8106022, 8222222, 8232383, 8546143, 8809292, 8828956, 9074213, 9370582, 9708610, 9708615, 10125369, 10131907, 10266825, 10273477, 10669544, 10806791, 10851377, 11078485, 11530408
214200	Cosela (trilaciclib)	8598186, 8598197, 9487530, 9957276, 10085992, 10189849, 10189850, 10927120, 10966984, 11040042, 11717523	11529352
214793	Pylarify (piflufolastat f 18)	8778305, 9861713, 10947197	8487129
215014	Empaveli (pegcetacoplan)	7888323, 7989589, 9169307	10035822, 10125171, 10875893, 11040107, 11292815, 11661441

© 2024 Ouellette LL et al. *JAMA Health Forum.*

eTable 4. FDA-Approved New Drug Applications with at Least One Bayh-Dole Patent in the Orange Book (editions 1985-2023), with the Share of Bayh-Dole Patents

NDA	Proprietary Name	% Bayh-Dole Patents
20162	ACTHREL (corticorelin ovine triflutate)	100
22549	ADASUVE (loxapine)	22
19937	ADENOCARD (adenosine)	100
21316	ALTOPREV (lovastatin)	33
213026	AMONDYS 45 (casimersen)	33
202008	AMYVID (florbetapir f-18)	100
21937	ATRIPLA (efavirenz; emtricitabine; tenofovir disoproxil fumarate)	25
208054	AXUMIN (fluciclovine f-18)	13
20727	BIDIL (hydralazine hydrochloride; isosorbide dinitrate)	33
210251	BIKTARVY (bictegravir sodium; emtricitabine; tenofovir alafenamide fumarate)	17
17954	BRETYLOL (bretylium tosylate)	100
22200	BYDUREON (exenatide synthetic)	3
21773	BYETTA (exenatide synthetic)	13
18874	CALCIJEX (calcitriol)	20
18312	CALDEROL (calcifediol)	50
19785	CARDIOLITE (technetium tc-99m sestamibi kit)	20
205494	CERDELGA (eliglustat tartrate)	29
19829	CERETEC (technetium tc-99m exametazime kit)	50
21197	CETROTIDE (cetrorelix acetate)	40
21673	CLOLAR (clofarabine)	100
202123	COMPLERA (emtricitabine; rilpivirine hydrochloride; tenofovir disoproxil fumarate)	21
214200	COSELA (trilaciclib dihydrochloride)	92
20869	COSOPT (dorzolamide hydrochloride; timolol maleate)	25
21366	CRESTOR (rosuvastatin calcium)	33
208215	DESCOVY (emtricitabine; tenofovir alafenamide fumarate)	50
18511	DRAXIMAGE DTPA (technetium tc-99m pentetate kit)	100
215014	EMPAVELI (pegcetacoplan)	33
21500	EMTRIVA (emtricitabine)	100

© 2024 Ouellette LL et al. *JAMA Health Forum.*

NDA	Proprietary Name	% Bayh-Dole Patents
21896	EMTRIVA (emtricitabine)	100
210951	ERLEADA (apalutamide)	40
206488	EXONDYS 51 (eteplirsen)	42
20044	EXOSURF NEONATAL (cetyl alcohol; colfosceril palmitate; tyloxapol)	100
210933	EYSUVIS (loteprednol etabonate)	100
22468	FOLOTYN (pralatrexate)	100
21481	FUZEON (enfuvirtide)	33
207561	GENVOYA (cobicistat; elvitegravir; emtricitabine; tenofovir alafenamide fumarate)	25
20919	GEODON (ziprasidone mesylate)	33
19863	GEREF (sermorelin acetate)	100
20443	GEREF (sermorelin acetate)	100
21335	GLEEVEC (imatinib mesylate)	25
21588	GLEEVEC (imatinib mesylate)	20
20637	GLIADEL (carmustine)	67
206073	GLYXAMBI (empagliflozin; linagliptin)	16
20076	HABITROL (nicotine)	67
20845	INOMAX (nitric oxide)	10
22037	INTUNIV (guanfacine hydrochloride)	33
210565	INVELTYS (loteprednol etabonate)	100
20084	IOBENGUANE SULFATE I 131 (iobenguane sulfate i-131)	100
21884	IPLEX (mecasermin rinfabate recombinant)	100
22044	JANUMET (metformin hydrochloride; sitagliptin phosphate)	38
202270	JANUMET XR (metformin hydrochloride; sitagliptin phosphate)	33
21995	JANUVIA (sitagliptin phosphate)	43
201281	JENTADUETO (linagliptin; metformin hydrochloride)	20
208026	JENTADUETO XR (linagliptin; metformin hydrochloride)	17
202343	JUVISYNC (simvastatin; sitagliptin phosphate)	38
21226	KALETRA (lopinavir; ritonavir)	38
21251	KALETRA (lopinavir; ritonavir)	43
21906	KALETRA (lopinavir; ritonavir)	32

© 2024 Ouellette LL et al. *JAMA Health Forum.*

NDA	Proprietary Name	% Bayh-Dole Patents
203414	KAZANO (alogliptin benzoate; metformin hydrochloride)	18
214012	LEQVIO (inclisiran sodium)	5
213378	LYBALVI (olanzapine; samidorphan l-malate)	23
202207	LYMPHOSEEK KIT (technetium tc-99m tilmanocept)	50
21446	LYRICA (pregabalin)	50
22488	LYRICA (pregabalin)	50
209501	LYRICA CR (pregabalin)	20
21674	MENOSTAR (estradiol)	67
21487	NAMENDA (memantine hydrochloride)	50
22271	NESINA (alogliptin benzoate)	25
204677	NEURACEQ (florbetaben f-18)	100
22325	NEXTERONE (amiodarone hydrochloride)	50
20659	NORVIR (ritonavir)	60
20680	NORVIR (ritonavir)	80
20945	NORVIR (ritonavir)	45
22417	NORVIR (ritonavir)	33
208351	ODEFSEY (emtricitabine; rilpivirine hydrochloride; tenofovir alafenamide fumarate)	31
207924	OLUMIANT (baricitinib)	20
210922	ONPATTRO (patisiran sodium)	8
213388	ORIAHNN (COPACKAGED) (elagolix sodium,estradiol,norethindrone acetate; elagolix sodium)	50
210450	ORILISSA (elagolix sodium)	46
217639	ORSERDU (elacestrant dihydrochloride)	29
22426	OSENI (alogliptin benzoate; pioglitazone hydrochloride)	17
19880	PARAPLATIN (carboplatin)	50
212937	PEDMARK (sodium thiosulfate)	25
20451	PHOTOFRIN (porfimer sodium)	83
21320	PLENAXIS (abarelix)	50
204442	PROBUPHINE (buprenorphine hydrochloride)	100
18936	PROZAC (fluoxetine hydrochloride)	20
214793	PYLARIFY (piflufolastat f-18)	75

© 2024 Ouellette LL et al. *JAMA Health Forum.*

NDA	Proprietary Name	% Bayh-Dole Patents
21964	RELISTOR (methylnaltrexone bromide)	11
208271	RELISTOR (methylnaltrexone bromide)	10
50790	RESTASIS (cyclosporine)	8
18044	ROCALTROL (calcitriol)	50
19608	SILDAFLO (silver sulfadiazine)	50
17381	SILVADENE (silver sulfadiazine)	100
215559	SOHONOS (palovarotene)	60
209531	SPINRAZA (nusinersen sodium)	36
209805	STEGLUJAN (ertugliflozin; sitagliptin phosphate)	38
203100	STRIBILD (cobicistat; elvitegravir; emtricitabine; tenofovir disoproxil fumarate)	21
19836	SUPPRELIN (histrelin acetate)	100
21746	SURFAXIN (lucinactant)	100
217171	SYFOVRE (pegcetacoplan)	30
210455	SYMTUZA (cobicistat; darunavir; emtricitabine; tenofovir alafenamide fumarate)	13
214460	TEMBEXA (brincidofovir)	100
214461	TEMBEXA (brincidofovir)	20
17675	TENATHAN (bethanidine sulfate)	100
20898	THYROGEN (thyrotropin alfa)	43
50753	TOBI (tobramycin)	100
208627	TPOXX (tecovirimat)	100
214518	TPOXX (tecovirimat)	100
201280	TRADJENTA (linagliptin)	20
22048	TRIESENCE (triamcinolone acetonide)	33
213436	TRUDHESA (dihydroergotamine mesylate)	67
20408	TRUSOPT (dorzolamide hydrochloride)	50
21752	TRUVADA (emtricitabine; tenofovir disoproxil fumarate)	43
19530	UCEPHAN (sodium benzoate; sodium phenylacetate)	100
19981	ULTRATAG (technetium tc-99m red blood cell kit)	100
209776	VABOMERE (meropenem; vaborbactam)	14
21267	VFEND (voriconazole)	29

© 2024 Ouellette LL et al. *JAMA Health Forum.*

NDA	Proprietary Name	% Bayh-Dole Patents
22253	VIMPAT (lacosamide)	100
22254	VIMPAT (lacosamide)	100
22255	VIMPAT (lacosamide)	100
21119	VISUDYNE (verteporfin)	9
203137	VIZAMYL (flutemetamol f-18)	60
210557	VYLEESI (AUTOINJECTOR) (bremelanotide acetate)	17
212161	VYNDAMAX (tafamidis)	67
211996	VYNDAQEL (tafamidis meglumine)	100
211970	VYONDYS 53 (golodirsen)	80
20597	XALATAN (latanoprost)	20
208400	XATMEP (methotrexate sodium)	20
214375	XENOVIEW (xenon xe-129 hyperpolarized)	100
211109	XERAVA (eravacycline dihydrochloride)	50
211950	XIPERE (triamcinolone acetonide)	33
203415	XTANDI (enzalutamide)	100
213674	XTANDI (enzalutamide)	100
20819	ZEMPLAR (paricalcitol)	60
21606	ZEMPLAR (paricalcitol)	100
209899	ZEPOSIA (ozanimod hydrochloride)	75
20412	ZERIT (stavudine)	100
20413	ZERIT (stavudine)	100
21453	ZERIT XR (stavudine)	50
20212	ZINECARD (dexrazoxane hydrochloride)	33
21991	ZOLINZA (vorinostat)	64

© 2024 Ouellette LL et al. *JAMA Health Forum.*
