# Supplementary material for: Using Bayh-Dole Act March-In Rights to Lower US Drug Prices
Source: JAMA Health Forum. 2024 Nov 1;5(11):e243775. doi: 10.1001/jamahealthforum.2024.3775 (PMC11530938; doi:10.1001/jamahealthforum.2024.3775)
Supplement: Supplement 2. — Data Sharing Statement [file jamahealthforum-e243775-s002.pdf]

Data Sharing Statement

Ouellette. Using Bayh-Dole Act March-In Rights to Lower US Drug Prices. *JAMA Health Forum*. Published November 01, 2024. doi:10.1001/jamahealthforum.2024.3775

Data
Data available: Yes
Data types: Data (not involving human participants)
How to access data: https://doi.org/10.7910/DVN/VI93T9
When available: beginning date: 02-29-2024

Supporting Documents
Document types: None

Additional Information
Who can access the data: All of the data is already publicly available under a CC0 1.0 (public domain) license.
Types of analyses: The data may be used for any purpose.
Mechanisms of data availability: All of the data is already publicly available under a CC0 1.0 (public domain) license.
